# TIN-X: target importance and novelty explorer

**DOI:** 10.1093/bioinformatics/btx200

**Published:** 2017-04-07

**Authors:** Daniel C Cannon, Jeremy J Yang, Stephen L Mathias, Oleg Ursu, Subramani Mani, Anna Waller, Stephan C Schürer, Lars Juhl Jensen, Larry A Sklar, Cristian G Bologa, Tudor I Oprea

**Affiliations:** 1 Translational Informatics Division, Department of Internal Medicine, University of New Mexico School of Medicine, Albuquerque, NM, USA; 2 UNM Center for Molecular Discovery, University of New Mexico Comprehensive Cancer Center, University of New Mexico, Albuquerque, NM, USA; 3 Department of Molecular and Cellular Pharmacology, Miller School of Medicine, University of Miami, Miami, FL, USA; 4 Novo Nordisk Foundation Center for Protein Research, Faculty of Health and Medical Sciences, University of Copenhagen, Copenhagen N, Denmark; 5 Department of Pathology, University of New Mexico, NM, USA

## Abstract

**Motivation:**

The increasing amount of peer-reviewed manuscripts requires the development of specific mining tools to facilitate the visual exploration of evidence linking diseases and proteins.

**Results:**

We developed TIN-X, the Target Importance and Novelty eXplorer, to visualize the association between proteins and diseases, based on text mining data processed from scientific literature. In the current implementation, TIN-X supports exploration of data for G-protein coupled receptors, kinases, ion channels, and nuclear receptors. TIN-X supports browsing and navigating across proteins and diseases based on ontology classes, and displays a scatter plot with two proposed new bibliometric statistics: Importance and Novelty.

**Availability and Implementation:**

http://www.newdrugtargets.org

## 1 Introduction

Science builds upon past discoveries, traditionally communicated through scientific literature. However, scale-out of traditional and alternate publication modes has exceeded the limits of human processing (Hunter and Cohen, 2006), creating the need for computer-assisted methods. Text mining, ontologies, and interactive visualization are some of the emerging technologies that can alleviate information overload. We present a new method and software tool utilizing these technologies, to provide biomedical scientists with rankings and visualizations linking proteins and diseases. The information is derived from PubMed abstracts, text mined using previously published tools for named entity recognition (NER) of gene/protein and disease names (Pletscher-Frankild *et al.*, 2015). We propose two new bibliometric indices based on NER mentions, specifically devised to address research planning use cases. Our goal is to enable scientists to identify research subjects with sufficient evidence of importance, but not already well-studied, with focus on target prioritization for drug discovery. *Novelty* estimates the scarcity of publications about a protein target (see also Equation 1). *Importance* estimates the strength of the association between that protein target and a specific disease (see also Equation 2). By visualizing Novelty and Importance on a 2D plot, users can easily examine the strength of the evidence linking protein targets and diseases of interest.

TIN-X is a web application which allows users to navigate targets via the drug target ontology (DTO, http://drugtargetontology.org) and diseases via the disease ontology (DO) (Kibbe *et al.*, 2015). In its current implementation, TIN-X allows users to query, browse and display disease-target associations for the following protein families: ion channels, G-protein coupled receptors (GPCRs), nuclear receptors (NRs) and kinases. These plots are interactive, with built-in drill-down and link-out functionality for in-depth examination of selected targets. TIN-X was inspired by and developed for the Illuminating the Druggable Genome (IDG) Knowledge Management Center (KMC, http://targetcentral.ws), which aggregates and integrates protein-centric information, and seeks to identify and prioritize understudied genes and proteins for further investigation and validation as potentially novel drug targets.

## 2 Methods and implementation

TIN-X is implemented in Scala using the Lift web framework (http://liftweb.net). The back-end utilizes a PostgreSQL database derived from the IDG-KMC knowledge base known as Target Central Research Database (TCRD) (Nguyen *et al.*, 2017). TCRD compiles data from over 50 genomic, proteomic and drug-centric sources, and relies on the Disease Ontology, as well as in-network (IDG-KMC) developed tools such as DTO and disease—genes/protein mapping using a highly efficient dictionary-based NER application (Pafilis *et al.*, 2013) with dictionaries from the DISEASES (Pletscher-Frankild *et al.*, 2015) database, respectively. Data are extracted, transformed, aggregated and loaded into TCRD, with routine updates quarterly. All PubMed abstracts are downloaded from NCBI FTP site. Bibliometric statistics and the derived Novelty (*N_i_*) and Importance (*I_ij_*) scores are pre-computed for runtime performance, using the following formulae:

(1) $$N_{i}=\;1/\sum_{k}\frac{1}{T_{k}}$$

(2) $$I_{ij}=\;\sum_{k}\frac{1}{T_{k}\cdot D_{k}}$$

where *T_k_* and *D_k_* are the numbers of targets and diseases in abstract (*k*), respectively, and summation over all publications including target (*i*), and for importance, also including disease (*j*). Fractional counts are employed to reflect strength of association. For example, if an abstracts mentions three targets once, each receives a fractional count 1/*T* = ⅓.

## 3 Application example

**Fig. 1:**
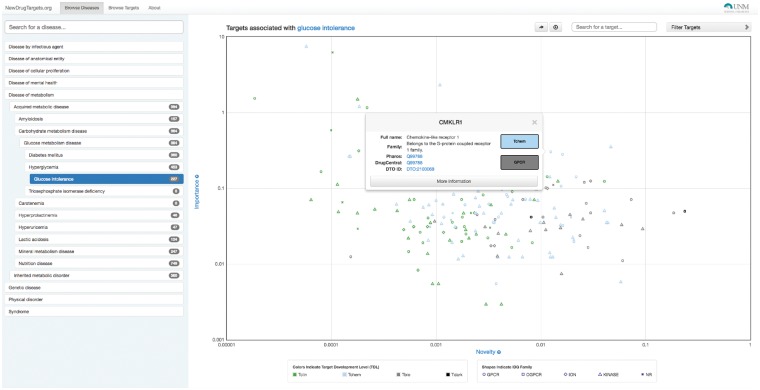
Screenshot of TIN-X. The disease, glucose intolerance, was queried. Mouse-over displays information about a specific data point, in this case, CMKLR1 (Color version of this figure is available at Bioinformatics online.)

**Fig. 2:**
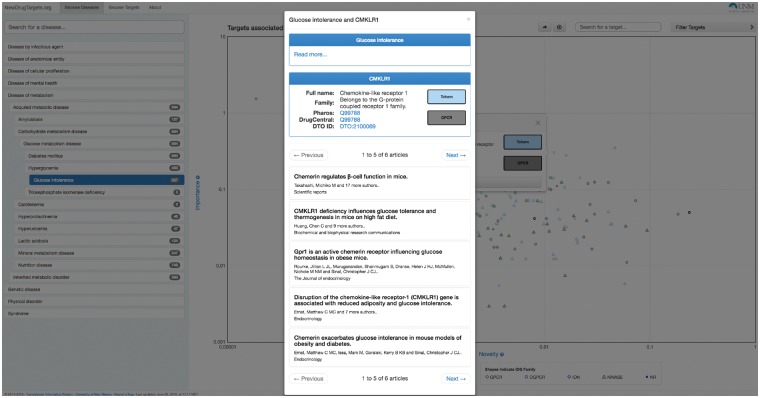
Screenshot of TIN-X. Click on selected point shows the papers where the target protein (CMKLR1) might be relevant to the disease (glucose intolerance)

In Figure 1, TIN-X displays targets for a selected disease, ‘glucose intolerance’. The DO hierarchy can be navigated in the left panel, and the targets are plotted with log–log Importance–Novelty axes. Searching by disease name and by target name (with auto-suggest) is supported. In the current layout, targets with stronger associations are in the upper part of the plot, while targets with a higher number of publications are on the left side of the plot. In general, more interesting associations will be on the upper right boundary of the plot, where data points represent non-dominated solutions to the multi-objective optimization maximizing both Importance and Novelty. Targets can be filtered based on Target Development Level, with Tclin representing mode-of-action drug targets (Santos *et al.*, 2017), and Tdark representing the understudied proteins (Nguyen *et al.*, 2017). Targets can be filtered by protein superfamily (e.g. GPCRs). Link outs to Pharos (targets) and DO (diseases) are provided for each association. Figure 2 illustrates the result of mouse-over actions for the target from Figure 1, ‘Chemokine-like receptor 1’ (CMKLR1), which links to 6 publications associating CMKLR1 and glucose intolerance. Titles and citations are displayed. Clicking on each article reveals full abstracts and links out to complete PubMed records, where users can examine the available evidence.

## 4 Conclusions and future directions

TIN-X provides an interactive visualization, ranking, and prioritization platform for scientists interested in exploring potentially novel drug targets, and examining the relationship between diseases, disease categories, proteins and protein classes, using automated text mining of biomedical literature. As with all current text mining, TIN-X cannot replace expert human readers and curators, yet, it is increasingly clear that automated bibliometry is essential given the accelerated pace and volume of publications. TIN-X is an important interactive tool in the IDG project, and future plans include adding functionality relating to known drugs and drug classes, and inclusion of additional protein classes. More information about Tclin, Tdark and other target development levels can be found at http://www.nature.com/nrd/posters/druggablegenome/

## Funding

This work was supported by the National Institutes of Health [U54 CA189205-01], and the Novo Nordisk Foundation [NNF14CC0001].

*Conflict of Interest*: none declared.
